# Supplementary material for: A tissue dissociation method for ATAC-seq and CUT&RUN in Drosophila pupal tissues
Source: Fly (Austin). 2023 May 21;17(1):2209481. doi: 10.1080/19336934.2023.2209481 (PMC10208176; doi:10.1080/19336934.2023.2209481)
Supplement: Supplemental Material [file KFLY_A_2209481_SM5227.docx]

**Supplemental Data and Protocols for**

**A tissue dissociation method for ATAC-seq and CUT&RUN in *Drosophila* pupal tissues.**

Elli M. Buchert ^1#^, Elizabeth A. Fogarty ^1#^, Christopher M. Uyehara ^2,3^, Daniel J. McKay ^2^ and Laura A. Buttitta ^1*^

1. Molecular, Cellular and Developmental Biology, University of Michigan, Ann Arbor 48109

2. Dept. of Biology, Dept. of Genetics, Integrative Program for Biological and Genome Sciences, University of North Carolina, Chapel Hill, Chapel Hill, NC 27955

3. Curriculum in Genetics and Molecular Biology, University of North Carolina, Chapel Hill, Chapel Hill, NC 27955

This Supplement contains:

Supplemental Fig. 1

Supplemental Fig. 2

Supplemental Fig. 3

Tables of reagents and detailed step-by-step dissociation protocol

Tables of reagents and buffers and detailed step-by-step protocol for OMNI-ATAC

Tables of reagents and buffers and detailed step-by-step protocol for Cut & Run


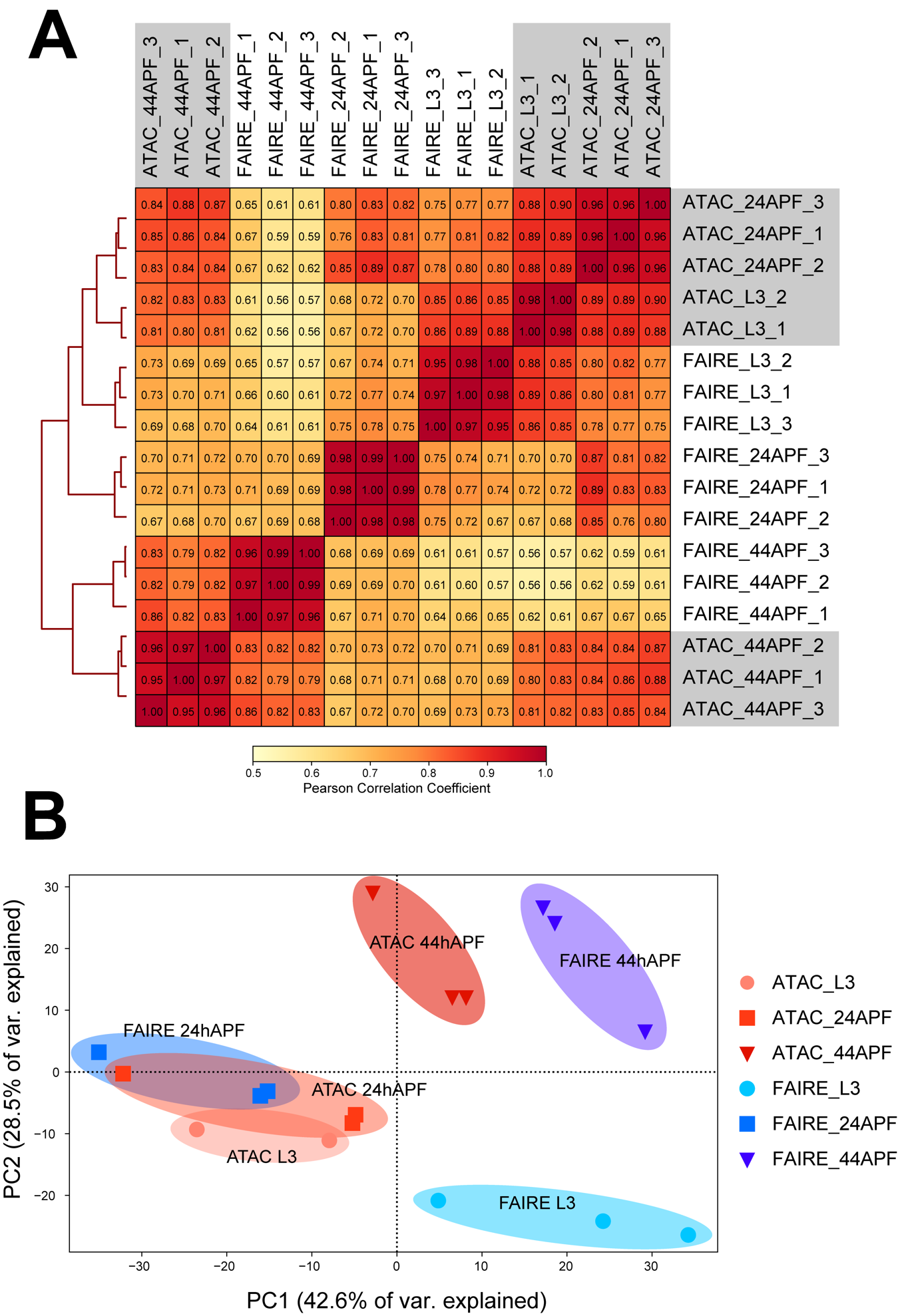


**Supplementary Figure 1. Pearson Correlation and PCA Analysis comparing ATAC-Seq and FAIRE-Seq data from larval and pupal wings.** Pearson correlation and hierarchical clustering analysis (**A**) and principal component analysis (**B**) of ATAC-Seq and FAIRE-Seq data from third instar larval (L3) wing discs and pupal wings at 24 and 44 hours after puparium formation (APF). Each analysis was performed based on read coverage from individual replicates within the union set of peak regions for all conditions.


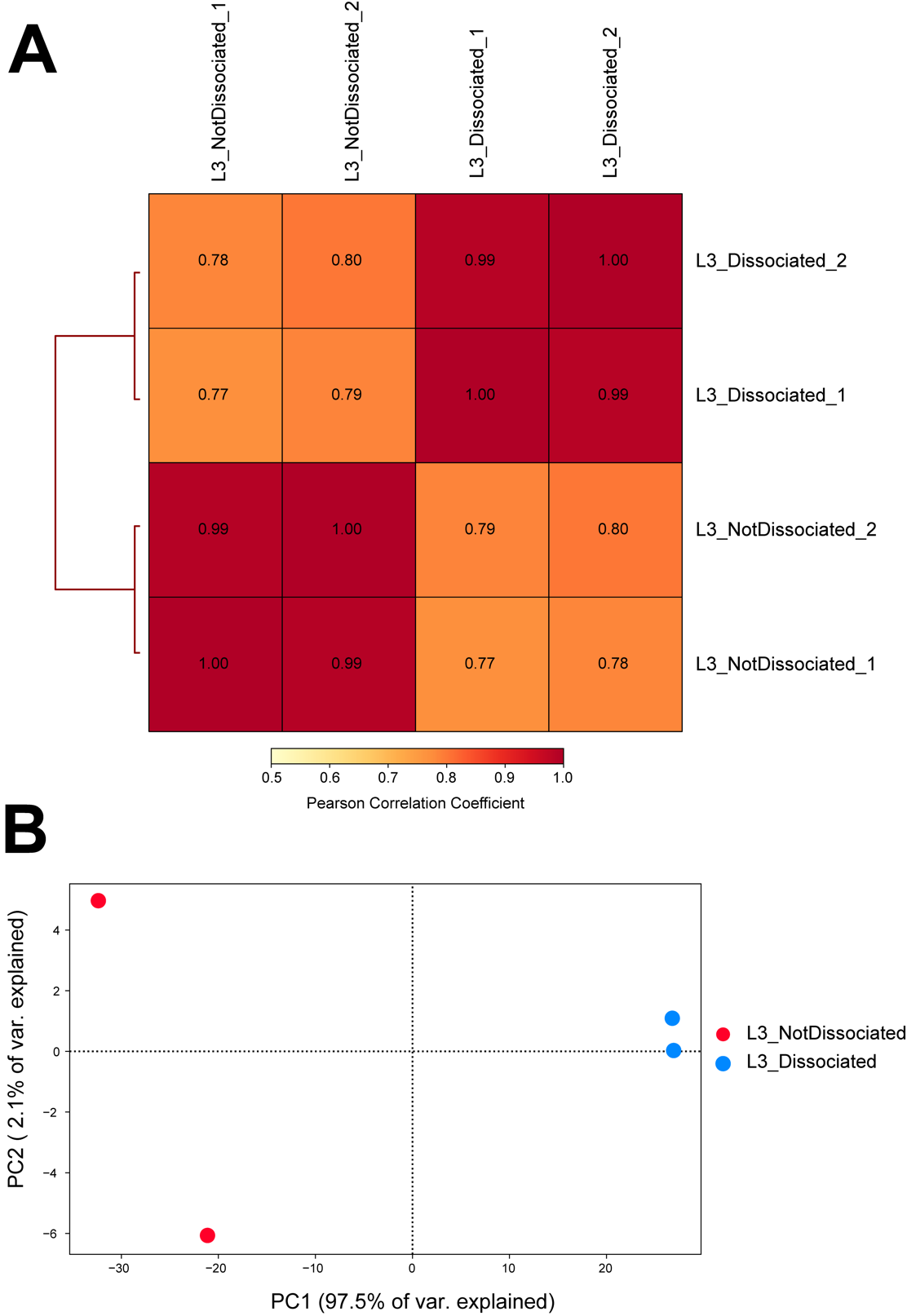


**Supplementary Figure 2. Pearson Correlation and PCA Analysis comparing ATAC-Seq data from dissociated and non-dissociated larval wing discs.** Pearson correlation and hierarchical clustering analysis (**A**) and principal component analysis (**B**) of ATAC-Seq data from third instar larval (L3) wing discs that were dissociated or not dissociated prior to cell lysis. Each analysis was performed based on read coverage from individual replicates within the union set of peak regions for both conditions.


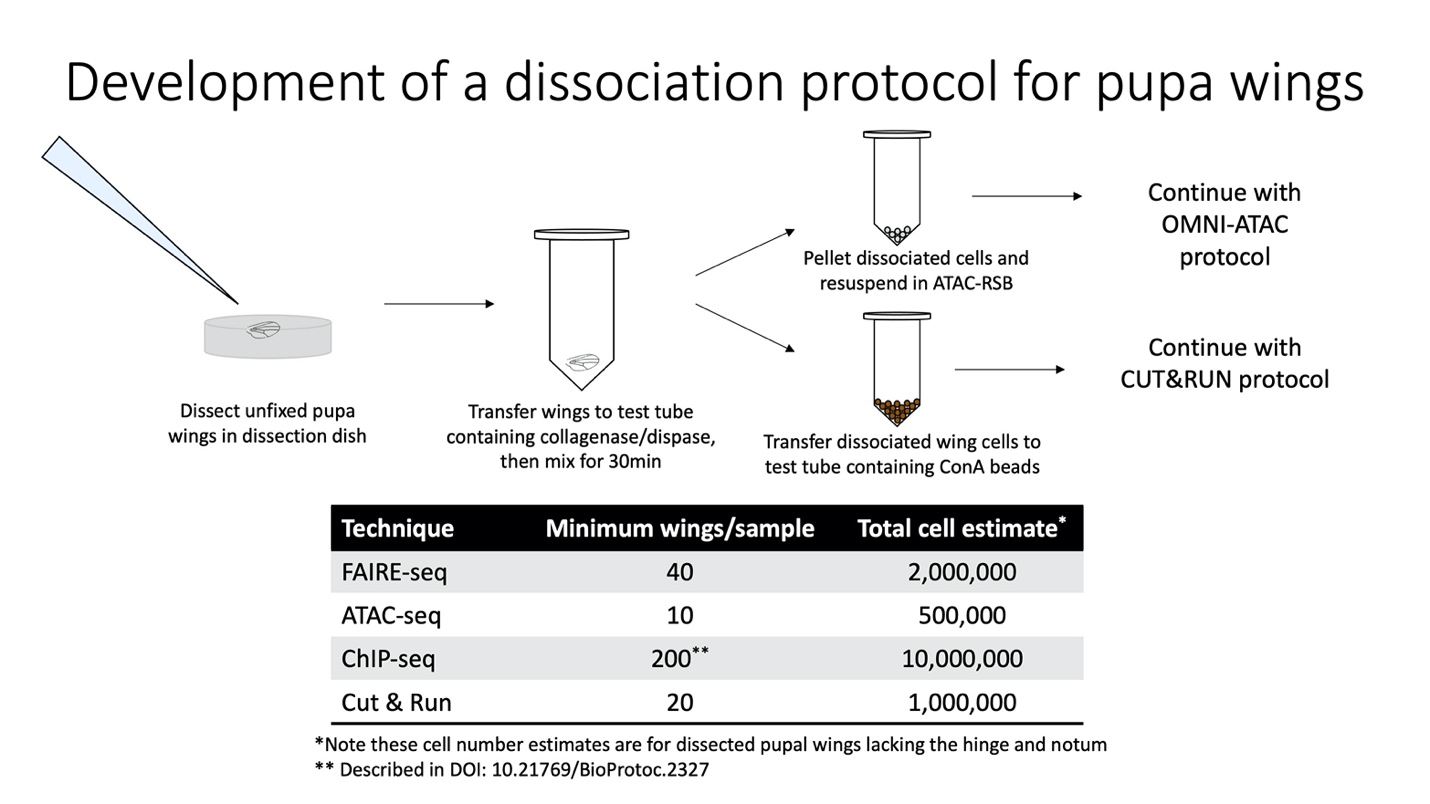


**Supplementary Figure 3. Protocol overview and cell number estimates.** The diagram outlines the steps for dissociation prior to ATAC-Seq or Cut & Run. The table provides estimates of the minimum number of wings and cells for each approach compared in this paper.

**Detailed Dissociation Protocol:**

Collagenase/Dispase Dissociation of Pupal Tissues for CUT&RUN or ATAC-seq

Reagents for Dissociation Protocol

| **Reagent** | **Supplier** | **Cat #** |
| --- | --- | --- |
| Collagenase/Dispase | Sigma | 10269638001 |
| HEPES | Fisher | BP310-500 |
| NaCl | Fisher | S271-500 |
| BSA | Fisher | BP1605-100 |
| Spermidine | Sigma | S0266-1G |
| Roche cOmplete EDTA-free protease inhibitor tablet | Sigma | 11873580001 |
| MilliQ Water |  |  |
|  |  |  |

Dissociation Step-by-Step Protocol:

1. Prepare 100mL of Wash Buffer (20mM HEPES pH7.5, 150mL NaCl, 0.1% BSA).
2. To make 50mL of Wash + Buffer: Add 0.5mM Spermidine and one Roche Tablet to 50mL of Wash Buffer.
3. Collagenase/Dispase comes as a 100mg lyophilized powder. Reconstitute in 1mL dH20 to make a 100mg/mL concentrated stock. Make 2X working stock to avoid repeated freeze-thaw cycles.
4. To make a working 2X stock (2mg/mL), dilute 100mg/mL concentrated Collagenase/Dispase 1:50 in HEPES pH 7.5 Wash Buffer. Make 100uL aliquots into 1.5mL Eppendorf tubes. Freeze concentrated and 2X working stocks at -20°C.
5. Dissect pupal tissues in cold Wash + solution.
6. Using a cut-off 200uL pipette tip pre-coated with fat body (to avoid tissue sticking), pipet tissues directly into 50-100uL 2X Collagenase/Dispase solution transferring minimal solution. For 10-20 wings, this usually transfers about 50-100uL of solution making the final concentration 1X.
7. Incubate at room temp for 30 min with shaking. We use an eppendorf thermomixer set to 23°C and 500 rpm.
8. Vortex for 10 sec at setting 6 (about 60% of max speed). Incubate another 10 min. with shaking. Vortex again for 10 sec at setting 6 (about 60% of max speed).
9. From here, proceed with ATAC-seq or CUT&RUN as you would with dissected larval discs in a similar volume.

**TABLES OF REAGENTS AND BUFFER COMPOSITIONS FOR ATAC-SEQ AND CUT & RUN**

Reagents for ATAC-seq

| **Reagent** | **Supplier** | **Cat #** |
| --- | --- | --- |
| Tris-HCl | Fisher | BP153-100 |
| Tween 20 | Fisher | BP337-100 |
| NP40 (IGEPAL CA-630) | Sigma | I8896 |
| Illumina Tagment DNA TDE1 Enzyme and Buffer Kits | Illumina | 20034197 |
| MinElute Reaction Cleanup Kit | Qiagen | 28204 |
| Sterile Water |  |  |

Reagents for CUT&RUN

| **Reagent** | **Supplier** | **Cat #** |
| --- | --- | --- |
| Concanavalin A (ConA) Beads | Polysciences | 86057-3 |
| Digitonin | Fisher | AC407565000 |
| Ampure XP Beads | Beckman Coulter | A63881 |
| 40% PEG 8000 | Sigma | P1458 |
| EDTA | Fisher | BP121-500 |
| EGTA | Sigma | E3889 |
| KCl | Fisher | P333-500 |
| CaCl_2_ | Sigma | C7902-500G |
| MnCl_2_ | Sigma | 244589-10G |
| 1M MgCl_2_ (RNAse, DNAse free) | VWR | 82023-086 |
| SDS | Acros Organics | 327311000 |
| RNAseA | Thermofisher | EN0531 |
| ProteinaseK | Thermofisher | EO0491 |
| SMARTer DNA Unique Dual Index Kit 24U Set | Takara | R400665 – R400668 |
| SMARTer ThruPLEX DNA-Seq Kit | Takara | R400674 – R400675 |

**Buffer Composition and detailed protocol for OMNI-ATAC:**

**Wash Buffer (store at 4C up to a month)**

20 mM HEPES, pH 7.5, NaOH (2 mL of 1M HEPES, pH 7.5)

150 mM NaCl (3 mL of 5 M NaCl)

0.1% BSA (0.1 g BSA)

95 mL autoclaved MilliQ H2O

**Wash+ Buffer (store at 4C up to a week)**

0.5 mM Spermidine (3.93 uL of d=0.925g/mL spermidine)

Roche Protease Inhibitor Tablet (1 per 50 mL)

50 mL Wash Buffer

**ATAC-RSB**

| Reagent | Final Concentration | Volume for 50 mL | Volume for 5 mL |
| --- | --- | --- | --- |
| 1M Tris-HCl pH 7.4 | 10 mM | 500 uL | 50 uL |
| 5M NaCl | 10 mM | 100 uL | 10 uL |
| 1M MgCl_2_ | 3 mM | 150 uL | 15 uL |
| Sterile Water | n/a | 49.25 mL | 4.925 mL |

**Detergents- 100X stock solutions**

Digitonin: 2% in DMSO. Dilute 1:1 with water to make 1% (100X stock). Avoid more than 5 freeze-thaws. Store at -20C up to 6 months.

Tween-20: 10% (100X stock). Store at 4C.

NP40: 10% (100X stock). Store at 4C.

**Protocol**

Before starting: set centrifuge to 4C.

1. **Dissection and Dissociation**
2. Dissect 10 wings or 16 eyes in Wash+ solution.
3. Pipette wings with cut-off P200 pipette tip into 100 uL of 2X Collagenase/Dispase solution transferring 100uL liquid.
4. Incubate on heat block set to 23C, rotating at 500 rpm for 30 minutes.
5. Vortex for 10 sec at setting 6 (60% of max).
6. Incubate on shaking block for 10 minutes.
7. Repeat 10 sec vortex.
8. Spin down at 800 x g, for 5 minutes, at 4C.
9. Remove supernatant and wash in 200 uL 1X PBS.
10. Repeat spin.
11. **Cell preparation**
12. Resuspend cell pellet in 50 µl cold ATAC-RSB supplemented with 0.1% NP40, 0.1% Tween-20, and 0.01% Digitonin. Pipette up and down 3 times. Incubate on ice 3 minutes.
13. Wash out lysis with 1 mL cold ATAC-RSB containing 0.1% Tween-20 but not NP40 or Digitonin. Invert tube 3 times to mix.
14. Spin down at 800 xg for 10 min, 4°C.
15. Discard supernatant and immediately continue to transposition reaction. Note that the pellet can be quite loose at this point. Often requires removing 1 mL of supernatant, then spinning down again for 5 min to re-pellet and remove the final 50 uL.
16. **Transposition reaction and purification**
17. To make the transposition reaction mix, combine the following:

25 µl 2x TD Buffer (Illumina Cat #20034197)

- 1. µl Tn5 Transposes (100nM final, Illumina Cat #20034197)

16.5 µl PBS

0.5 uL 1% Digitonin

0.5 uL 10% Tween-20

5 uL water

1. Resuspend nuclei in the transposition reaction mix.
2. The transposition reaction is carried out at 37°C for 30 min shaking at 1000 RPM.
3. Following transposition reaction, the sample is purified using a Qiagen MinElute kit.
4. Elute transposed DNA in 21 µl Elution Buffer (10 mM Tris buffer pH 8.0).
5. Purified DNA can be stored at -20°C. (Typically takes about 3 hours from starting dissection to this point.)
6. **PCR Amplification**
7. To amplify transposed DNA fragments, combine the following in a PCR tube. Include a no-template control (NTC).

20 µl transposed DNA

2.5 µl Nextera fw PCR primer, 25uM (general fw primer)

2.5 µl Nextera rev PCR primer, 25uM (specific rev primer, contains barcode)

25 µl NEBNext High-Fidelity 2x PCR Master Mix

1. Cycle as follows:
2. 72°C, 5 min
3. 98°C, 30 sec
4. 98°C, 10 sec
5. 63°C, 30 sec
6. 72°C, 1 min
7. Repeat steps 3-5, 5x
8. Hold at 4°C
9. In order to reduce GC and size bias in PCR, the PCR reaction is monitored using qPCR to stop amplification prior to saturation. To run a qPCR side reaction, put PCR reactions on ice and combine the following. Include the NTC.

5 µl 5 cycles PCR amplified DNA

3.76 µl Nuclease Free H_2_O

0.5 µl Nextera fw PCR primer, 25uM (general fw primer)

0.5 µl Nextera rev PCR primer, 25uM (specific rev primer, contains barcode)

0.24 µl 25X SYBR Green dye

5 µl NEB Next 2X Master Mix

1. qPCR cycle as follows:
2. 98°C, 30 sec
3. 98°C, 10 sec
4. 63°C, 30 sec
5. 72°C, 1 min
6. Repeat steps 3-5, 20x
7. Hold at 4°C
8. The additional number of cycles needed for the remaining 45 µl PCR reaction is determined as following:
9. Plot linear Rn vs. Cycle
10. Set 5000 RF threshold
11. Calculate the # of cycle that is corresponded to ¼ of maximum fluorescent intensity

*NTC sample should not amplify. Libraries should need very few, if any, additional cycles using this protocol.

1. Run the remaining 45 µl PCR reaction to the correct # of cycles. Cycle as follows:
2. 98°C, 30 sec
3. 98°C, 10 sec
4. 63°C, 30 sec
5. 72°C, 1 min
6. Repeat steps 2-4, x times (determined above)
7. Hold at 4°C
8. Purify amplified library using SPRI beads as follows:
   1. Add 0.5X volume (22.5 uL) of beads to each sample. Pipette 10X to mix.
   2. Incubate at RT 10 minutes.
   3. Place tubes on magnetic rack 5 minutes.
   4. Transfer supernatant to new tube.
   5. Add 1.3X original volume (58.5 uL) of beads. Pipette 10X to mix.
   6. Incubate at RT 10 minutes.
   7. Place tubes on magnetic rack 5 minutes.
   8. Discard supernatant.
   9. Wash beads with 200 uL fresh 80% EtOH. Pipette EtOH over beads 10X. Discard EtOH.
   10. Incubate on magnet with lids open 10 minutes. Monitor to ensure that beads are dry but not cracked.
   11. Resuspend beads in 20 uL H2O. Pipette 10X to mix.
   12. Place tubes on magnetic rack 1-5 minutes.
   13. Transfer supernatant to new tube.
   14. Store libraries at -20C.

**Buffer Composition and detailed protocol for Cut & Run:**

**Solutions**

**Make Ahead**

Solutions Required (make all with Autoclaved MilliQ water):

1M HEPES, pH 7.9 (pH with KOH)

1M HEPES, pH 7.5 (pH with NaOH)

1M KCl

0.5M MnCl2

1M EDTA

100mM EGTA (must pH above 8.0 with NaOH to get EGTA into solution)

100mM CaCl2

10% SDS

RNAseA 10mg/ml (-20C)

ProteinaseK 20mg/ml (-20C)

5% Digitonin in DMSO (weigh out with full PPE & double gloves, good for 3 months at -20C)

| Binding Buffer (keeps at 4C for 6months):  20 mM HEPES-KOH, pH7.9 (1ml of 1M HEPES, pH 7.9)  10 mM KCl (0.5ml of 1M KCl)  1 mM CaCl2 (0.5ml of 100mM CaCl2)  1 mM MnCl2 (0.1ml of 0.5M MnCl2)  47.9ml Autoclaved MilliQ water | Wash Buffer (store up to a month 4C)  20 mM HEPES, pH7.5, NaOH (2ml of 1M HEPES, pH 7.5)  150mM NaCl (3ml of 5M NaOH)  0.1% BSA (0.1g BSA)  95ml Autoclaved MilliQ water |
| --- | --- |
| Wash + (store 1 week 4C)  0.5mM Spermidine (3.93ul of d = 0.925g/ml Spermidine)  Roche Tablet (1 per 50ml)  Wash Buffer (50ml) | DBE (Digitonin Block EDTA) (store O/N 4C)  2.00 mM EDTA (30ul of 1M EDTA)  0.1 % Digitonin (0.3ml of 5% Digitonin)  15.00 ml Wash + |
| 2xSTOP  200 mM NaCl (0.4ml of 5M NaOH)  20 mM EDTA (0.2ml of 1M EDTA)  4 mM EGTA (0.4ml of 100mM EGTA)  9ml Autoclaved MilliQ water | Digitonin Buffer (store O/N 4C)  0.1 % Digitonin (20ul of 5% Digitonin)  1.00 ml Wash + |
| MXP Buffer (Magnesium ampureXP buffer)  20.0 % PEG8000 (2ml of 40% PEG8000)  2.5 M NaCl (2ml of 5M NaCl)  10.0 mM MgCl2 (RNAse, DNAase Free)(40ul of 1M MgCl2) | HXP Buffer (Homemade ampureXP buffer)  20.0 % PEG8000 (1ml of 40% PEG8000)  2.5 M NaCl (1ml of 5M NaCl) |

**Make During Protocol**

| 2x Reaction Buffer (75 uL / sample)  1500.0 uL Wash+  60.0 uL 100mM CaCl_2_ | Pellet Buffer (150uL per sample)  250.00 uL Wash +  250.00 uL 2xSTOP  5.31 uL SDS (10%)  6.89 uL proteinase K (20mg/mL) |
| --- | --- |
| 2xSTOPyR  500.00 uL 2xSTOP  3.13 uL RNAseA (10mg/ml) |  |

**_______________________________________________________________________**

**Day 1:**

Collect Tissue:

1. Collect & sort larvae/pupa in dissection dish in PBS
2. Dissect larvae/pupa in **Wash+** buffer in dissection dish, at room temp (**RT**)
3. Transfer wings to dish w/ ~**200uL** **Wash+** in well **on ice**
   1. Transfer individual wings using forceps or p200 w/ pipette tip cut off
   2. If pipetting, be careful about wings sticking to pipette tip
4. If using pupa wings, see Pupa Wing Dissociation protocol

Prepare ConA Beads:

1. Place on nutator at RT for **30min** prior to use
2. Transfer **15 uL** **ConA** beads per sample to Eppendorf (**not** low retention, beads will stick to tubes).
3. Bind to magnet **5min**
4. Remove Supernatant
5. Add **1mL** **Binding buffer**
   1. Incubate **1 min**.
6. Wash **2 X** **1ml binding buffer**
7. Resuspend **15 uL Binding buffer** per sample

Bind Discs to magnet:

1. Add **15 uL Bead slurry** to Eppendorf per sample
2. Transfer discs in minimal volume (~**80 uL**) to **bead slurry** using cut-off p200 pipette tip
   1. Mix w/ gentle pipetting
3. Add **1mL** **DBE**, pipette to mix
   1. Incubate **10min on ice**
4. Make antibody dilutions in **DBE** (**100uL per sample)**
5. Antibody Dilution: Here we used Rabbit Anti-H3K27me3 (C36B11) mAb cat # 9733 from Cell Signaling at a 1:100 dilution
6. Bind discs to magnet **2min**
   1. Remove buffer, replace with **100uL** **antibody soln**
   2. Incubate sample angled sideways on orbital shaker **O/N @ 4°C (Walk In)**

**Day 2:**

pA-MNase Digestion: *(performed at 4°C)*

1. *Optional: remove liquid from top of tube, if necessary*
   1. *Quick spin (<1s)*
2. Remove Binding Buffer
   1. Add discs to magnet
      1. Incubate **2min, 4C**
   2. Wash **2 X 500uL DBE**, invert back & forth ~10x to mix
      1. Incubate **2min, 4C**
   3. Transfer to magnet
3. *Optional: Prepare secondary antibody soln in* ***DBE (100 uL / sample)***
   1. Antibody Dilution:
   2. Incubate **1 hr, 4C**
   3. Repeat washes (**2x500uL DBE**; 2m, 4C between washes)
4. Prepare pA/G-MNase dilution in **DBE** (**100 uL / sample**)
   1. **Dilute EpiCypher pA/G-MNase 1:20 into DBE, keep on ice**
5. Bind samples to magnet **2min**
   1. remove buffer, resuspend in **100 uL pA-MNase soln**
   2. incubate **10 min @ RT** orbital shaker angled
6. Make **2x Reaction Buffer, 2x STOPyR Buffer and Pellet Buffer**:
   1. For STOPyR and Pellet Buffer, add RNase/Proteinase K/SDS ~5 min before use
   2. Chill in ice bath
7. Bind samples to magnet x 2min, remove sup
   1. Wash **500 uL** **Wash+**, resuspend, incubate **2min**
   2. Repeat 1x
8. Bind sample to magnet
   1. Add sample, **incubate 2min**
   2. Remove supernatant, resuspend discs in **75 uL** **Digitonin Buffer**, pre-chill tubes on ice
   3. Add **75 uL** **2xRxn Buffer** to samples
      1. Digest for **2 hours @ 4C** on orbital shaker
   4. Add **150 uL** **2xSTOPyR** to stop rxn, pipette to mix

**Fragment Release:** *(Remainder of protocol performed at room temp)*

1. Incubate samples **30m, 37C**
2. Bind sample on magnet **2min**
   1. ****KEEP SUPERNATANT**** Transfer supernatant to low retention tube labeled “S”
   2. Resuspend pellet in **150 uL** **Pellet Buffer** (label “P”)
3. Add **2 uL 10% SDS** and **2.5 uL proteinase K (20mg/mL)** to supernatant samples
   1. mix by briefly vortexing
   2. Incubate supernatant and pellet samples **50°C, 2hr** (**or O/N**)
   3. With **30 min** remaining, place **Ampure XP** beads on nutator at RT to resuspend

DNA Recovery

1. P tubes on magnet **2min**
   1. ****KEEP SUPERNATANT**** transfer supernatant to new low retention tube
   2. To new tube add **40uL AmpureXP** beads and **65 uL** **HXP** buffer
      1. Final Vol ~**255 uL** (0.7x ratio)
   3. mix by pipetting, 10x
   4. incubate **15min, RT**
2. P tubes on magnet **5min**
   1. transfer supernatant to new low retention tube (“**PX**”)
   2. add **40uL AmpureXP beads** and **155 uL MXP** (**final ~ 450 uL**: 2x ratio)
   3. mix 10x pipetting
   4. incubate **15min, RT**
3. S tubes:
   1. add **40 uL** **AmpureXP** beads and **560 uL** **MXP** (**final ~900 uL** [2x ratio])
   2. mix 10x by pipetting,
   3. incubate **15min,** **RT**
4. S and PX tubes:
   1. Bind beads to magnet **5min**
   2. Make 80% EtOH **fresh** from 100% EtOH. Be sure to accurately measure H2O volume w/ pipette
   3. *Carefully* remove supernatant
   4. Keeping tubes on magnet, add **1mL 80% EtOH** to each tube, incubate **30sec**
   5. repeat 1x
   6. Thoroughly remove EtOH, leaving
      1. leave ~**70 uL**; pulse spin (<100rpm < 1s); magnet 1min, remove remaining
   7. Air dry **3min**
      1. **Do NOT** over dry! Resuspend samples before beads begin to “crack”--prefer slightly wet beads.
5. Resuspend beads in **14uL** Nuclease-free water
   1. Incubate **10min, RT**
   2. Bind beads **5min**
   3. Transfer **2 x 6uL** with p10 to fresh, low retention tube
      1. Using p10 makes it easier to avoid beads
      2. store samples at -20 °C or <2 weeks at 4C
   4. Remove remaining **1-2uL** from beads to Qubit rxn
      1. don’t take beads!
